# Supplementary figures and images for: dGPredictor: Automated fragmentation method for metabolic reaction free energy prediction and de novo pathway design
Source: PLoS Comput Biol. 2021 Sep 27;17(9):e1009448. doi: 10.1371/journal.pcbi.1009448 (PMC8496854; doi:10.1371/journal.pcbi.1009448)

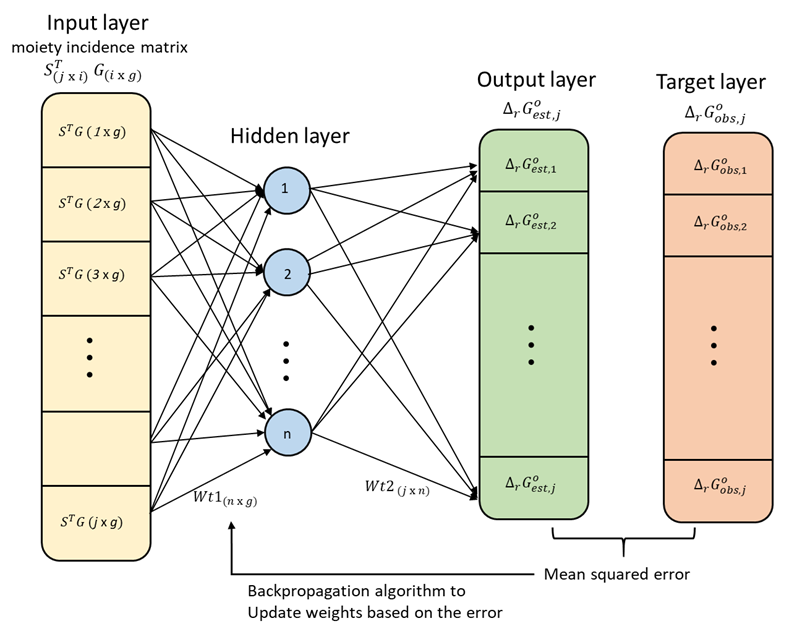

Supplement: S1 Fig — An input layer of moiety incidence matrix (j x g matrix), a hidden layer with n neurons, and an output layer of estimates with j neurons. Here j represents the reactions, g represents the groups/moieties, and n represents the neurons in the hidden layer. (TIF) [file pcbi.1009448.s007.tif]
