# Supplementary material for: dGPredictor: Automated fragmentation method for metabolic reaction free energy prediction and de novo pathway design
Source: PLoS Comput Biol. 2021 Sep 27;17(9):e1009448. doi: 10.1371/journal.pcbi.1009448 (PMC8496854; doi:10.1371/journal.pcbi.1009448)
Supplement: Attachment — Submitted filename: dG_reviewer_replies_v0.docx [file pcbi.1009448.s008.docx]

**Comments to the Authors:
Please note here if the review is uploaded as an attachment.**

**Reviewer #1:** The paper by Wang et al. describes a new method for estimating Gibbs energy changes of chemical reactions. In comparison with previously developed Group/Component Contribution methods where molecules were decomposed into manually selected functional groups, Wang et al. describe an approach where they split a molecule into chemical moieties with distance up to 2 bonds. They performed regression analysis based on the experimental data from a thermodynamic database, obtaining a model that is able to predict Gibbs energy change of a reaction with high accuracy. This approach allows to include stereochemistry in the energy estimation, something that was not achieved with previously developed methods. Overall the paper describes a useful methods, is well written and I would recommend it for publication after several major and minor points aare addressed.

**Major points:**

1. While the authours do provide a convenient browser-based tool to calculate the Gibbs energy of a reaction, they do not provide an intuitive Python tutorial to calculate Gibbs energies for a set of reactions. One clear jupyter notebook with the same example as shown in the Demo gif on github would suffice this purpose. This is a crucial step for the method to be used by the community.

– Following the reviewer's suggestion, a stepwise tutorial on implementing dGPredictor using Streamlit is provided. It is available as Readme.MD File in the Github codebase.

2. Explanation related to Figure 4D at lines 326 - 339 is flawed. The authors explain that moiety C[C(@ or @@)H](O)(O) is not chiral in RDKit due to absence of explicit protons at C atom. This is wrong. The moiety is not chiral because in this SMILES notation it's attached to two oxygen atoms. Since there are no branching after both oxygens the C atom in this moiety is not stereogenic. I do agree that if one looks for connectivity over two bonds (M2) then this moiety is chiral, but the explanation should be corrected. Additionally, RDkit does provide an option to draw hydrogen atoms explicitly (see [https://www.rdkit.org/docs/GettingStartedInPython.html#modifying-molecules](https://nam10.safelinks.protection.outlook.com/?url=https%3A%2F%2Fwww.rdkit.org%2Fdocs%2FGettingStartedInPython.html%23modifying-molecules&data=04%7C01%7Cvuu10%40psu.edu%7Cb4424cb8772e430158d408d8fbaa824a%7C7cf48d453ddb4389a9c1c115526eb52e%7C0%7C0%7C637536057044351436%7CUnknown%7CTWFpbGZsb3d8eyJWIjoiMC4wLjAwMDAiLCJQIjoiV2luMzIiLCJBTiI6Ik1haWwiLCJXVCI6Mn0%3D%7C1000&sdata=5sLa%2FrQL9MyR%2FTsfDVm3zgRSsEF3jD0IImHG5%2F%2Be4jE%3D&reserved=0)).

– We thank the reviewer for pointing out that the moiety is not chiral for distance one due to two oxygen atoms attached to the carbon atom. Therefore, the reaction does not cause any moiety changes. The explanation is now updated in the paper as "RDkit cannot identify the chiral center when the "C-O-H" and "C-O-" bonds are responsible for tetrahedral stereochemistry, leading to the same SMILES string for different moieties and a loss of stereochemical information for reactions using M1 moiety change. An example is shown in Fig 4D, where KEGG reaction R10764 that converts $\alpha$-L-Fucopyranose to $\beta$-L-Fructopyranose which is expected to have non-empty moiety changes if the moieties preserve the same stereo configurations (i.e., [C@H] or [C@@H]) as in the molecular structure of $\alpha$-L-Fucopyranose and $\beta$-L-Fructopyranose for bonding distance one. In this case, RDkit returning an empty group can be alleviated by combining both distances to capture a non-zero moiety change." (Line 336-344 in the revised manuscript)

3. Authors do not mention anything about reactions including Fe or polymeric structure (starch, glycogen etc.). How well does their methods handle such compounds?

– The training data does not have any instances involving Fe or polymeric structures. In principle, the methodology is open to use other heteroatoms, assuming that there is a dataset to train. This has now been clarified in the manuscript. (Line 489-492)

**Minor points:**

1. In the first paragraph/sentence of the introduction, the authors motivate their work, primarily from a metabolic engineering point of view. However, thermodynamics have been used in the context of metabolism in a much broader context. Thus, by placing their work in a bigger context, they could further increase the impact of their work.

– We agree with the reviewer's comment that the prediction of free energy of reactions is a task that cuts across many disciplines beyond metabolic engineering. In the updated manuscript, we broadly cite a number of calculation tasks that require accurate $\Delta_{r}G^{o}$ of reactions and a short description (Line 50-55). However, dGPredictor was trained using metabolic reactions; therefore, it is uncertain how it will perform for general chemistries.

2. In the 3rd sentence of the introduction, it sounds like beta-oxidation is an engineered pathway.

– Beta-oxidation is a native pathway, but it has been engineered before to operate in the reverse direction [1]. This is clarified in the revised manuscript. (Line 58-59)

3. Could authors add a short comment regarding bias of predictions towards the compounds in the database. For example, I imagine there many more L-amino acids than D-amino acids in the database. How would it affect the predictions for D-amino acids?

– The automated moiety generation method uses the SMILES notation to represent the stereochemistry in the molecules. An analysis for reactions with no-group changes has been provided to show dGPredictor's ability to capture stereochemistry. We found that the MSE of the reactions involving no-group changes is 5.06 compared to the GC method with MSE 63.97, which cannot discriminate reactions that only undergo stereo-specific change. Since dGPredictor has the ability to discriminate the moieties generated for L- and D-amino acids, we do not think there will be any bias caused due to the number of L- and D-amino acids present in the database. (Line 326-330)

Could the authors specify units in the equation at Line 285 and check if units for sigma (J/mol) are correct? I suspect it should be kJ/mol. Also provide units at Line 291.

– Yes, the reviewer is correct. The units are indeed kJ/mol. This has been corrected in the revised manuscript.

5. One of the revisions of the Equilibrator included additional information on interaction with Mg2+. It's known that this ion affects thermodynamics of many biochemical reactions due to binding. Do the authors consider influence of Mg?

– We did not consider the influence of Mg because Du et al. [2] pointed out that the accuracy of the $\Delta_{r}G^{o}$ estimates decreased when Mg influence estimates were used due to limited available data for ion-binding constants. Nevertheless, in the latest version, the Equilibrator tool [3] uses the ion-binding constants predicted from Du et al. and some other experimental measurements to capture the influence of Mg. This data were not available to us at the time of building the dGPredictor tool.

6. I wonder if it's possible to decompose the confidence interval of the Gibbs energy of a reaction into a standard deviation obtained from the regression and a standard deviation obtained for unknown moieties (33.29 (k?)J/mol).

– We use the Scikit-learn python package for performing the regression to estimate the Gibbs energy contribution of moieties. The tool Sklearn directly provides the confidence intervals of the Gibbs energy of reaction and the standard deviation in the moieties as an output [4].

7. The eQuilibrator website has turned into THE source for the community. I would strongly encourage that the authors of this paper here get in touch with the people behind the eQuilibrator to see whether the new code could be implemented into the eQuilibrator. Otherwise, I fear that the community, even though the data/tool developed here is superior to what is behind the eQuilibrator, will continue to use eQuilibrator.

– We thank the reviewer for this comment. We plan to reach out to the creators of eQuilibrator upon completion of the review process of this manuscript.

8. In the discussion the authors mention that the new tool can now be used to predict for which reactions/compounds we would need measurements to further improve the predictions. I would greatly appreciate if the authors could add such analysis such that they could add some concrete suggestions on what we as a community should measure.

– A list of reactions has been included and provided in supplementary file S5 Table for which the $\Delta_{r}G^{o}$ cannot be estimated with accuracy due to the lack of moieties present in the compounds in the TECRDB database that are needed to describe metabolites in the KEGG database. This list was ranked by the number of unmeasured moieties in the reactions. Thus, it can be used to prioritize experiments to focus on reactions for which experimental $\Delta_{r}G^{o}$ measurements can help improve accuracy of the tool. A brief note on this has been added in the revised article. (Line 459-462)

9. According to the IUPAC suggestions, the term "free" should be dropped in Gibbs FREE energy, see [https://goldbook.iupac.org/terms/view/G02629](https://nam10.safelinks.protection.outlook.com/?url=https%3A%2F%2Fgoldbook.iupac.org%2Fterms%2Fview%2FG02629&data=04%7C01%7Cvuu10%40psu.edu%7Cb4424cb8772e430158d408d8fbaa824a%7C7cf48d453ddb4389a9c1c115526eb52e%7C0%7C0%7C637536057044351436%7CUnknown%7CTWFpbGZsb3d8eyJWIjoiMC4wLjAwMDAiLCJQIjoiV2luMzIiLCJBTiI6Ik1haWwiLCJXVCI6Mn0%3D%7C1000&sdata=igRjWqcOHGuQHEKPIo6V6DMd0tUO2CPQ9yWxso6N9vQ%3D&reserved=0). Thus, I suggest that the authors only use "Gibbs energy".

– Following the reviewer's comment, the word "Free" has been dropped from "Gibbs free energy" throughout the paper.

10. In some parts of the manuscript the authors use GC (group contribution) when referring to the work of Noor et al, and later they use CC (component contribution). It would be great if the authors could make this consistent.

– Following the reviewer's comment, we have made it consistent by using GC throughout the paper.

11. Line 187-188: I didn't fully get this sentence.

– Line 187-188 "We chose to assess moieties associated with a bonding distance one and two to select the model with better prediction accuracy."

This describes how different models (i.e., using bonding distance of one and two) were built, and the model with better prediction accuracy was chosen as the best model for dGPredictor. This clarification has been included in the text (lines 186-188).

12. Lines 218-22"" Is there an intuitive explanation for this finding? If there is, maybe the authors could hint to this?

Lines 218-22 "Similar to the linear models, $\Delta_{r}G^{o}$ prediction accuracy (i.e., a lower MSE value) improved with increasing bonding distance (see Table 2). Models M2-nonlinear and M1,2-nonlinear were nearly identical in their prediction accuracy within the training datasets (MSE 6.92 vs. 6.95) and outperformed the M1-linear model (>3-fold higher MSE 20.81). The LOOCV MAE was lower for the M1,2-nonlinear model than the M2-nonlinear (7.27 vs. 14.69, see Table 2), indicating that the former is the best-performing nonlinear model."

– The intuitive explanation here is that the results are similar to the linear model case. The MSE decreases as we increase the number of moieties with distance two due to overfitting. The M1,2-nonlinear model performs well in terms of both MSE and cross-validation score as it selectively picks the best moieties out of both distance one and two to classify molecules. (Line 214-222)

13. Line 243-244: I didn't get it. Also, I think the regularization appears here for the first time. It would be good if the authors could introduce this before; what do you put in the regularization term?

Line 243-244 "dGPredictor mitigates this issue by applying regularization in the regression analysis."

–The regularization term is provided in the Method section. A brief explanation is now added in the text to briefly explain how regularization is used to resolve the issue. (Line 241-244) **- "**dGPredictor mitigates this issue by applying regularization in the regression analysis, which shrinks the moiety contribution estimates by adding L2-regularization term in the regression objective function which also helps avoid overfitting.**"**

14. Comparison of lines 260 and 268: Here, I am confused (but maybe I miss a point). In line 260, it is stated that dGPredictor can cover all metabolites; while in line 268 it is stated that only 69% of all reactions can be estimated. What is the reason that even though that all metabolites can be described we still cannot parametrize all reactions?

– The experimental data available for compounds in TECRDB is not sufficiently diverse to cover all the compounds in the KEGG database. Thus, in order to parameterize all reactions in the KEGG database, we used Bayesian ridge regression (Line 289-297) and moieties from KEGG compounds to train the model (see table 3)

15. Lines 286-288: Please add some more explanation.

"After the hyperparameter optimization during model training (see Methods section 'Bayesian ridge regression to determine the Gibbs free energy of groups $\Delta_{g}G^{o}$'), maximizing the log of the posterior distribution in Bayesian ridge regression yields the optimized precision parameters $\alpha$ as $9.023\cdot10^{-4}$."

– Following the reviewer's comment, an explanation is provided on the hyperparameter optimization. (Line 288-290)

– " After the hyperparameter optimization during model training (see Methods section ‘Bayesian ridge regression to determine the Gibbs energy of groups $\Delta_{g}G^{o}$’) to estimate parameter $\alpha$ [26] by maximizing the log of the posterior distribution in Bayesian ridge regression yields a parameter $\alpha$ of $9.023 10^{-4}$."

16. Line 329: Word missing?

– We thank the reviewer for pointing out a missing subject in the sentence. It has been corrected in Line 334

17. Line 183: Gibb's

– Following the reviewer's comment, the formatting has been corrected

18. Figure 4B is mentioned in the text before Figure 4A.

– Following the reviewer comment, the sequence in the figure is updated

19. Line 386: Group change or moiety change?

– It is moiety change, and it has been made consistent throughout the paper

**Reviewer #2:** In this work, Wang et al. present their automated method for metabolite decomposition and show how it can be applied within a framework similar to group contribution to predict standard GIbbs free energies. Using a standard function such as "FindAtomEnvironmentOfRadiusN" from RDkit is a very elegant solution and indeed expert-curated functional group definitions are no longer needed, group contribution-based methods would benefit greatly in terms of simplicity and generalizability.
The text is well-written, in a clear language, and the flow is logical and coherent.

I do, however, have major concerns regarding the analysis of the method and they way it was compared to previously published works on this topic:

* Line 192: "Model prediction accuracy" is defined as the MSE over the entire dataset. Since this is not a cross-validation error, I disagree that it represents prediction accuracy, but rather the goodness of fit of the model to the training data. Comparing this measure between algorithms that have very different degrees of freedom is quite misleading and should at least be corrected using the AKAIKE information criterion. In any case, the cross-validation prediction accuracy score is the *only* valid way to compare prediction performance and referring to it as "overfitting potential" seems to understate its real value. The fact that GC performs best among all tested methods is a very big caveat to this work and the authors must address this point and convince the readers how the other advantages of their method could counterweigh this disadvantage.

– MSE is provided as a metric for the goodness of fit in prediction over training data and Cross-validation median MAE as a metric to evaluate both prediction accuracy and overfitting. We chose these metrics so a direct comparison with the state-of-the-art algorithm eQuilibrator can be made [6]. Since both GC and dGPredictor has comparable performance in cross-validation, a better goodness of fit and the ability of dGPredictor to resolve major limitations of covering KEGG reactions and considering stereochemistry makes it a superior tool compared to group contribution.

* Line 439: "MAE … was comparable to the current state-of-the-art CC method …". However, it seems that the comparison was done with the less advanced "group contribution" method. If this is indeed the case, why was the new method not compared to the state of the art (e.g. the method by Du et al. from 2018)? The authors should add this to the comparison table, or give reasons why this is not a fair comparison (and change the text to clearly say what was it compared to).

- The reason was that the method by Du et al. [2] have performed 10-fold cross-validation in their study and compared the results with widely known Noor's group contribution method [6]. The method by Du et al. included the influence of temperature and Mg ions on the $\Delta_{r}G^{o}$ predictions, which increase the median MAE of the GC method. However, they used a different dataset that contains 432 overlapping reactions from the previous GC and 185 new reactions curated in the paper. Therefore, to directly compare with the widely known GC method, we only use the data present in Noor's GC, i.e., from the NIST TECRDB database [7] using the same leave-one-out cross-validation and same prediction accuracy metric as Noor's GC method.

One of the discussed advantages of the proposed method is the ability to more accurately predict non-zero energies for isomerization reactions. Although it sounds reasonable, there is no real data to back this claim. We are only given one example (which could also just be a coincidence). A more thorough analysis of all such reactions, e.g. in the form of a cross-validated mean error, would be necessary to support the current claim.

The example is provided to demonstrate that it is possible to capture free energy of change for reactions with no group change using moieties. In the paper, we have provided an analysis that infers 319 KEGG reactions with no group changes issue, a list of all such reactions, and corresponding experimental $\Delta_{r}G^{o}$ is provided in supplementary file S3 Table. In addition, we provide the mean squared error for just the reactions with no-group changes for both GC and dGPredictor, in order to demonstrate that it is not a coincidence that the reaction with zero group change has non-zero Gibbs energy and the MSE value for GC method for no-group change reaction is 63.97 compared to 5.06 of dGPredictor. Furthermore, the cross-validation for only those reactions also shows a similar trend as the mean error for the GC method is 3.84 and dGPredictor is 2.26. (Line 326-330)

* Although highlighted in the title of this paper, the thermodynamic tool for de novo pathway design and the text describing it are not well-developed. First of all, it's not very clear what the novelty of this tool is (besides the fact that it uses the dGPredictor library). In fact, more advanced methods for thermodynamic feasibility of designed pathways are available for many years (e.g. [https://doi.org/10.1093/bioinformatics/bti213](https://nam10.safelinks.protection.outlook.com/?url=https%3A%2F%2Fdoi.org%2F10.1093%2Fbioinformatics%2Fbti213&data=04%7C01%7Cvuu10%40psu.edu%7Cb4424cb8772e430158d408d8fbaa824a%7C7cf48d453ddb4389a9c1c115526eb52e%7C0%7C0%7C637536057044361428%7CUnknown%7CTWFpbGZsb3d8eyJWIjoiMC4wLjAwMDAiLCJQIjoiV2luMzIiLCJBTiI6Ik1haWwiLCJXVCI6Mn0%3D%7C1000&sdata=1WxlDAPF8D7JNB4aZ2AYUbYRHt43F5te5D58TIZb8EY%3D&reserved=0)) and it seems that the current implementation is a step backward in terms of sophistication. The way irreversible reactions are defined is wrong (the standard ΔG'0 doesn't have to be both positive and negative within the confidence interval, but rather based on concentration ranges). The naive criterion of |ΔG'0| > 20 kJ/mol can quite often lead to mistakes, and the authors should consider replacing it with the established methods.

- The paper listed by the reviewer uses the group contribution method [8]. dGPredictor allows for broader reaction coverage of reactions for which fragmentation using groups is impossible, or the same groups are generated for both reactants and products. dGPredictor can distinguish between stereoisomers and estimate $\Delta_{r}{G'}^{o}$for reactions that involve novel molecules. The moiety change matrix can be used for both $\Delta_{r}{G'}^{o}$ evaluation and de novo pathway design, which allows consistent integration of thermodynamic analysis with pathway design tool instead of using two separate tools. The reviewer is correct that the actual concentrations would ultimately determine the Gibbs energy of the reaction. However, actual concentrations are rarely known during the pathway discovery stage. We used the conservative cutoff of |ΔG'0| > 20 kJ/mol as a threshold for deciding on whether to allow a reaction to be treated as reversible. Reversing reactions with a $\Delta_{r}{G'}^{o}$ > 20 kJ/mol would require highly imbalanced concentrations that are unlikely to be physiologically relevant. Of course, a user could invoke different criteria for deciding on reversibility. This is clarified in the revised manuscript. (Line 395-401)

* Although I do see the potential in a tool such as the one shown in figure 5, which could be an interesting addition to the existing ones, I was not able to evaluate it due to technical reasons (see Minor point #2). That being said, even if the software would work, it's not clear what the scientific novelty of it would be. It seems more as a software integration issue and probably doesn't belong in this manuscript at all.

– Following the reviewer comment, the GitHub repository has been updated and a stepwise tutorial has been provided for the software as a README.md file on GitHub.

**Minor points:**

* When adding new compounds using InChI, how are the proton dissociation constants obtained for it? ChemAxon Marvin is mentioned once in the manuscript but it doesn't seem to be used anywhere in the scripts. Without these pKa values, any estimation of reactions with novel compounds would not be valid.

– See Lines 571-573; the proton dissociation constant was obtained using the ChemAxon command-line tool "cxcalc". We use a function from the Component contribution method [6] to estimate the pKa values. The dependency has now been provided in the "README.md" file on GitHub.

* In line 179: "to standardize the experimental values … at pH 7 and ionic strength 0.25 M". This is not correct, as the inverse Legendre Transform converts the biochemical Gibbs energies to "chemical" energies, where pH and ionic strength are undefined.

– Following the reviewer's comment, the sentence has been corrected as "to standardize the experimental values to a single pseudoisomer, we use inverse Legendre transformation to reduce the ensemble of pseudoisomers to the most abundant pseudoisomer at pH 7 and ionic strength 0.25M."

* I was not able to run the code stored on GitHub. Each of the Jupyter notebooks fails to run until the end due to missing files: "./data/median_b_extended.json" needed in the Jupyter notebook nalysis_dGPredictor.ipynb does not exist, "./model/bayesianRL_ac_all_model.sav" required for analyze_prior.ipynb is missing and so is "data/reaction_rule_r2_py3_manual_modified.csv" which is needed in new_linear_combine.ipynb. In addition, the Streamlit webapp didn't work, probably due to specific versions required for some of the dependencies (there is no requirements.txt or setup.cfg file as is standard in python packages). These technical issues should all be solved before the manuscript is published.

– Following the reviewer's comment, the missing files have been uploaded, and a stepwise tutorial has been provided for running dGPredictor locally using streamlit in README.md file on GitHub.

* The added value of figure 7 is not clear, as it shows a rather standard MLP network. Given the poor results, it seems unnecessary to dedicate a whole figure to it.

– Following the reviewer's comment, the figure has been moved to supplementary file S6 Figure.

**References**

[1]	C. Dellomonaco, J. M. Clomburg, E. N. Miller, and R. Gonzalez, "Engineered reversal of the β-oxidation cycle for the synthesis of fuels and chemicals," *Nature*, vol. 476, no. 7360, pp. 355–359, Aug. 2011.

[2]	B. Du, Z. Zhang, S. Grubner, J. T. Yurkovich, B. O. Palsson, and D. C. Zielinski, "Temperature-Dependent Estimation of Gibbs Energies Using an Updated Group-Contribution Method," *Biophys. J.*, vol. 114, no. 11, pp. 2691–2702, Jun. 2018.

[3]	M. E. Beber, M. G. Gollub, D. Mozaffari, K. M. Shebek, and E. Noor, "eQuilibrator 3.0-a platform for the estimation of thermodynamic constants," 2021.

[4]	F. Pedregosa FABIANPEDREGOSA *et al.*, “Scikit-learn: Machine Learning in Python Gaël Varoquaux Bertrand Thirion Vincent Dubourg Alexandre Passos PEDREGOSA, VAROQUAUX, GRAMFORT ET AL. Matthieu Perrot,” 2011.

[5]	M. Tipping, "SparseBayesianLearningandtheRelevanceVector Machine."

[6]	E. Noor, H. S. Haraldsdó ttir, R. Milo, and R. M. T Fleming, "Consistent Estimation of Gibbs Energy Using Component Contributions," *PLoS Comput Biol*, vol. 9, no. 7, p. 1003098, 2013.

[7]	R. N. Goldberg, Y. B. Tewari, and T. N. Bhat, "Thermodynamics of enzyme-catalyzed reactions-a database for quantitative biochemistry," *J. Phys. Chem-ical Ref. Data*, vol. 20, no. 16, p. 16, 2004.

[8]	V. Hatzimanikatis, C. Li, J. A. Ionita, C. S. Henry, M. D. Jankowski, and L. J. Broadbelt, "Exploring the diversity of complex metabolic networks," *Bioinformatics*, vol. 21, no. 8, pp. 1603–1609, Apr. 2005.
