# Supplementary material for: dGPredictor: Automated fragmentation method for metabolic reaction free energy prediction and de novo pathway design
Source: PLoS Comput Biol. 2021 Sep 27;17(9):e1009448. doi: 10.1371/journal.pcbi.1009448 (PMC8496854; doi:10.1371/journal.pcbi.1009448)
Supplement: Attachment — Submitted filename: replies_reviewers_comment2_8-16_final.docx [file pcbi.1009448.s009.docx]

**Comments to the Authors:
Please note here if the review is uploaded as an attachment.**

Reviewer #1: The authors have addressed our minor comments sufficiently. Our main concerns remain: their code implementation (Major point #1 previously) and in part their reply to Major point #2.
Code (Major point 1):
1) The author's choice to do their work in Python 2.7 is problematic because it is deprecated since 01.01.2020. Even some dependencies that the authors use (i.e. steamlit) are no longer supported, which makes the installation guide that they provided for the installation unusable. We could not execute it. This problem must be fixed.

- Following the reviewer's comments, the dGPredictor tool is now changed to python 3 and available on GitHub. It has been tested on multiple platforms running Windows and MacOS (https://github.com/maranasgroup/dGPredictor)

2) Second, while the web interface might be suitable to look up values for a couple of reactions, we also wondered about a more convenient way to "bulk-retrieve" data for many reactions, as it might be required for genome-scale models.

- Following the reviewer's comments, a jupyter notebook "retrieve_bulk.ipynb" is provided to retrieve $\Delta_{r}{G'}^{o}$ for a list of reactions in the KEGG database on our GitHub page (https://github.com/maranasgroup/dGPredictor)

Major point #2:
We think that the author's explanation is still somewhat incomplete with regards to this point. The authors state: "… This is because the RDkit tool used for computing moieties stores molecules with implicit hydrogen (i.e., not explicitly present in the molecular graph) and *ignores the "C-H/O-H" bond when* generating the SMILES string. Thus, RDkit cannot identify the *chiral center when the "C-O-H" and "C-O-" bonds are responsible* for tetrahedral stereochemistry". What is confusing is that the authors say "C-H, O-H are ignored", but then the authors mention the problem of a chiral center with C-O and C-O-H. In fact, chirality is also lost in SMILES notation for other bonds, e.g. C-C bonds, as in the example with 3-methyhexane CH3 - CH2 -CH (CH3) - CH2 - CH2 -CH3 (Smiles: CC(C)CCCC). The third C atom from the left is only chiral when you consider the full molecule. When you look at M1 and M2 distances as authors do, this atom is not chiral in SMILES notation.

- The reviewer is correct in the provided example. The explanation is now revised to clarify that RDkit cannot distinguish an atom to be chiral unless all the atoms are different at the specific bonding distance being considered. We have used moieties of up to distance two (i.e., M2), and it would indeed require moieties of bonding distance three to capture the chirality in the provided example. However, we found that moieties associated with longer bonding distances tend to significantly increase in number and cause overfitting for Gibbs energy estimates (Line 338-345).

Reviewer #2: I'm afraid that the answers that the authors provided to my comments are not always sufficient.

- Indeed, dGPredictor significantly increases the coverage compared to previous methods, but the abstract claims "has a higher prediction accuracy as compared to existing GC methods...". At least according to table 2, this is an incorrect statement (the cross-validation MAE is slightly larger).
Please change this statement to be correct (comparable cross-validation performance, better goodness-of-fit and coverage). I would even drop the claim for better goodness-of-fit since it only indicates that the system is over-trained.

- Following the reviewer's comment, the abstract has now been revised to state that both the GC method and dGPredictor have comparable prediction accuracy.

- Regarding the comparison with Du et al., the fact that it is easier to compare to, doesn't justify using the older method. In fact, the 185 new curated reactions could highlight the advantage of dGPredictor even further (since the new algorithm has more degrees of freedom and therefore gain more from the new data). It is curious to me that the authors chose to ignore Du's method because it "increase[s] the median MAE of the GC method", since that increase was very small (and comparable to the increase found in this study as well).

- We believe that implementing the groups adopted in the Du et al. study goes beyond the scope of this paper. The goal in this study is not to exhaustively assess every possible group contribution method but rather to highlight the advantages of using moieties in place of molecular groups for Gibbs energy prediction and offer an easy to use interface.

- The data regarding reactions with no group change seems quite strange. GC should always predict a zero reaction energy change (maybe slightly shifted do to the pKas), and therefore the MSE should simply be the variance of the ΔG0 data for these reactions. This should also be the case when doing cross-validation (since we don't actually use the linear model for this prediction). If this is the case, how can the MSE be 63.97 kJ/mol (much higher than the mean across all reactions in general - which is by itself not very likely) while the cross-validation MAE is only 3.84 kJ/mol (much lower than the average)?

- We agree with the reviewer's comment. GC does predict a zero $\Delta_{r}G^{o}$ and the MSE of 63.97 kJ/mol is the variance of all $\Delta_{r}G^{o}$ for the reactions with zero group change in the training data. However, we use mean absolute error for cross-validation, i.e., the actual $\Delta_{r}G^{o}$ for every iteration of LOOCV, which is the reason for the low mean for the cross-validation score compared to the MSE. An excel sheet (Supplementary Table S3) has been provided for reactions with no group change and corresponding ΔG for training and validation datasets. For validation data, the reaction duplicates are removed and replaced with the median ΔG for each reaction. The reviewer can verify that in Table S3, we list the cross-validation using both mean squared error and MAE. The mean score for the two methods is 48.20 (kJ/mol)^2^ and 3.84 kJ/mol, respectively. Hence the score using MSE in cross-validation is close to the MSE for fitting compared to MAE.

In addition, the abstract claims that the proposed method is an improvement because it adds consideration of stereochemistry within metabolite structures. However, this claim is not tested directly, but only together with the use of the graph-based groups (instead of user defined ones). If stereochemistry is important - it can also be added to the user-defined groups. Perhaps doing that would create a better result than dGPredictor?

- It is plausible that adding user-defined groups accounting for stereochemistry can be added in the user-defined list of groups to capture no-group change reactions. However, this would amount to over 163 groups only for the TECRDB database. Furthermore, GC would still be limited in scope when considering novel reactions. The automated moiety generation framework presented here allows the inclusion of stereochemistry within the molecular structure. It thus provides an advantage in estimating Gibbs energy compared to employing user-defined groups.

- The definitions and names in the manuscript are not very clear. I understand that dGPredictor is a catchy name for the code package, but it probably should be separated from the method itself. Using NovoStoich to replace the user-defined groups in GC is the main novelty and the name dGPredictor does not reflect that (i.e. it is too generic). In addition, the paper seems to throw in other tools that fit in the dGPredictor software suite, but are not very relevant to this work. I pointed out before that the tool for testing the reversibility of reactions is an inferior algorithm to other existing tools (I'm not going to repeat what I wrote here), and it seems that the only reason to mention it is its reliance on the dGPredictor library. It is my hope (and apparently also that of the other reviewer) that dGPredictor could be integrated into other existing software packages, so that its advantages would be easily accessible to the community. If the authors insist on keeping the dual focus (both on the algorithm itself, and on the software package), then they must improve the coding standards (see my last comment).

- NovoStoic is a pathway design tool, whereas dGPredictor is a framework for estimating $\Delta_{r}G^{o}$ of reactions. dGPredictor replaces the user-defined groups (as defined in the current state-of-the-art GC method) with chemical moieties. The estimated dG from dGPredictor can be used in novoStoic (or other retrosynthesis tools) to ensure thermodynamic feasibility of reaction direction for individual reaction steps in the pathway. The paper listed by the reviewer for testing the reversibility uses the group contribution method [1]. The dGPredictor allows for (i) broader reaction coverage of reactions for which fragmentation using groups is impossible, (ii) can distinguish between stereoisomers, and (ii) estimate $\Delta_{r}{G'}^{o}$for reactions that involve novel molecules. (See response to reviewer 1)
- The package has now been ported to Python 3 (https://github.com/maranasgroup/dGPredictor). Detailed scripts are provided to enable using dGPredictor to determine Gibbs energy for reactions and integrate pathway design tools such as NovoStoic. We have made the web-based interface to facilitate easy access to estimate Gibbs energy, and the method can be easily integrated with other software packages using the python functions in the "retrieve_bulk.ipynb" jupyter notebook provided in the GitHub repository.

- A lot of the values in the text are missing units (mostly kJ/mol or kJ^2/mol^2). Here are a two out of the many other cases: Line 203, Table 2.
It's not clear why the fit is described by a Mean squared error, while the cross-validation is using Mean absolute error (although in figure 2 the median is shown with the same values, is it Mean or Median??). RMSE (Root Mean Squared Error) is the most common measure and can be applied in both cases (it was used to compare the last 6 predictors in the Du et al. review paper - DOI:10.1016/j.tibs.2018.09.009). In general, the cross-validation error should be higher than the fitting error and it's hard to see it when the metrics are different (also, see my comment about the no-group-change reactions).
Can the authors explain why GC has by far the worst MSE but also the best cross-validation MAE? Is it an indication that all other methods are more susceptible to over-training?

- Following the reviewer's comment, we have fixed all the missing units in the paper.
- We use MSE over training data and MAE for cross-validation to directly compare with previous methods that have used the same metric to show model performance [2]. The worst MSE is due to fewer unique groups than chemical moieties generated in dGPredictor. In table 2, all other dGPredictor models are susceptible to over-training as they have more moieties than groups in the GC method. Therefore, we have used the cross-validation score to measure the over-fitting potential of these models generated for dGPredictor. A model with lower cross-validation error shows better performance in terms of over-training, leading us to pick the M1,2-linear model for dGPredictor.

- Please improve the coding standards of your software package. Python 2.7 is unsupported since January 2020 ([https://www.python.org/doc/sunset-python-2/](https://nam10.safelinks.protection.outlook.com/?url=https%3A%2F%2Fwww.python.org%2Fdoc%2Fsunset-python-2%2F&data=04%7C01%7Cvuu10%40psu.edu%7Cdd7ebc87feff4b81686208d9421db44d%7C7cf48d453ddb4389a9c1c115526eb52e%7C0%7C0%7C637613517609519512%7CUnknown%7CTWFpbGZsb3d8eyJWIjoiMC4wLjAwMDAiLCJQIjoiV2luMzIiLCJBTiI6Ik1haWwiLCJXVCI6Mn0%3D%7C1000&sdata=YIrMjMobemA7%2BzHSK1g%2Bxt2eh7jCnOuu28qE%2F9T13gU%3D&reserved=0)). There are countless tools that can help one port the code to Python 3. Downloading a .zip file from a sharepoint folder cannot be part of the installation process - and completely defeats the purpose of hosting code on GitHub (it seems to be only relevant for the comparative analysis, so probably optional - it would be best to mention that and make sure it's not necessary). In addition, the README still has some errors. For example, the first step is: Generate model file by "running model_gen.py" using "python model_gen.py". However, the file model_gen.py is not in the repository. The next step (running streamlit) did not work on my computer either. It could have to do with different platforms, but I urge the authors to try and facilitate running their tool on more platforms.

- Following the reviewer's suggestion, the package is ported to python 3 and all the relevant files are made available to our GitHub page. The package has been tested on multiple platforms such as Windows and Mac. In addition to that, we provided a jupyter notebook "retrieve_bulk.ipynb" for retrieving the Gibbs energy change estimation for a list of KEGG reactions (https://github.com/maranasgroup/dGPredictor).
